# Exploring research trends and priorities of genus *Melia*

**DOI:** 10.1038/s41598-024-53736-3

**Published:** 2024-03-15

**Authors:** Suresh Ramanan S, Ayyanadar Arunachalam, Uttam Kumar Sahoo, Kalidas Upadhyaya

**Affiliations:** 1 ICAR-Central Agroforestry Research Institute, Jhansi, 284003 India; 2 Department of Forestry, School of Earth Sciences and Natural Resource Management, Mizoram University, Aizwal, 769004 India

**Keywords:** *Melia*, Timber, Secondary metabolites, Topic modelling, Science mapping, Wood, Forestry, Tropical ecology, Ecology, Ecology

## Abstract

The genus *Melia* is known for its secondary metabolites and recently, this genus is being explored for its timber. There are vast differences among its species. For instance, *Melia azedarach* is reported to be invasive and while another species, *M. dubia,* has diverse utility with complex germination and regeneration characteristics. Researchers globally have been working on various aspects of this genus; In this study, using topic modelling and science mapping approach, we attempted to understand research facets on this genus. The literature corpus of the Web of Science database was explored using a single keyword—“Melia” which yielded 1523 publications (1946–2022) and after scrutiny metadata of 1263 publications were used in the study. Although nine individual species were cited in the publications, only three species are accepted viz., *M. dubia, M. azedarach,* and *M. volkensii*. This implies taxonomic uncertainty, with potential confusion in assigning scientific findings to particular species. Thus, a taxonomic relook on this genus is warranted for a better assessment of the economic utility in many countries. More importantly, our results indicate that the research interests have recently shifted from the secondary metabolite constituents towards growth, biomass, wood properties, germination, plantation, and green synthesis. The shift in research focus toward wood properties of *Melia* sp. can impact the wood demand–supply at a global scale owing to its fast growth and the possibility of cultivation over a wider geographical range.

## Introduction

Tree species of Meliaceae are known for their timber and Meliaceae family is also known as Mahagony family. The name is based on the Mahogany (*Swietenia* sp.) wood in well-known in international trade since 1900s^1^. Out of the 51 genera in the family, the important timber genera are *Entandrophragma*, *Khaya, Cedrela*, *Toona*, *Chukrasia*, *Azadirachta*, *Dysoxylum*, *Soymida*, *Xylocarpus*, and *Melia*^2^. Genus *Melia* has a trans-Indian Ocean distribution and its native to South Asia including the Indian sub-continent, northern Australia, and southern parts of tropical Africa. Yet there are fossil reports of this genus in the Americas and other countries leading to the conclusion that this genus might have diffused westwards in Miocene (23.03–5.33 Ma)^3^. It is closely associated to the genus *Azadirachta* and both of these genera belong to the tribe Melieae^4^. Across time, the members of the genera *Melia* and *Azadirachta* have been studied for their phytochemical/ secondary metabolite components which are used in ethnobotanical and pharmacological medicines^5^*.*

One of the common species this genus is *Melia azedarach* L*.* which is well known for its medicinal attributes in Chinese medicine^6^. It is regarded as the source of highly effective and low-toxic broad-spectrum plant-derived pesticides^7^. Yet, this species is also considered to be in invasive^8^. Recently, there is growing interest another species of this genus—*Melia dubia* Cav., a fast-growing species in India^9^. Other prominent members of this genus are *Melia composita* Benth., *Melia volkensii* Gürke*.*, etc. In India, the forests are protected since the 1980s for ecological purposes (protective services) and so, wood or timber demand is mainly met from the Trees Outside Forests (ToF) including the trees on farmlands (ToFa). The initiatives promoting commercial cultivation of tree species has limited to a few species like Poplar, Eucalyptus, Teak, Casuarina, etc. Lately, the indigenous, fast-growing tree *M. dubia* (known as ‘Malabar Neem’) has captured the attention of the stakeholders in India owing to its spreading crown and a cylindrical clean straight bole of about 9–10 m. Similarly in the Philippines, *M. dubia* is being explored for its timber utility with the aim to substitute it for prominent timber species like *Falcataria moluccana, Eucalyptus deglupta, Acacia mangium,* and *Swietenia macrophylla*^10^. In Africa, even though *Melia azedarach* is reported to be invasive South Africa, while *M. volkensii* has been reported for diverse utility in Kenya, Tanzania, and Somalia. Also, *M. volkensii* is being propagated via vegetative propagation owing to its complexity in germination and regeneration^11^. Earlier days, all *Melia* sp. were known for their secondary metabolites and ethnomedical purposes^12,13^. There is a shift and *Melia* sp. is being explored for timber utility. Overall, these members of this genus have distinct variations and characteristic differences in their economic utility. Since researchers are working on different dimensions of the genus *Melia*, the present work is aimed at exploring how research on the genus has evolved across priorities temporally.

In this study, we explore the research trend on this genus using the topic modelling and science mapping approaches. These two approaches aims at broad synthesis and this methodology will indicate the knowledge gaps and clusters, thereby enabling furture works on the identified topics^14^. The development of the research area is objectively assessed using the topic modelling approach to identify the emergent themes and topics in the body of literature through algorithms^15^. And science mapping is used to display the structural and dynamic aspects of scientific literature and thereby represent a cognitive structure of the research field per se*.* This will provide a holistic view of a particular field of science, and to identify key areas of research for further exploration. Using these approaches, the member of *Melia* sp. known for multiple uses including timber was explored to decide on the way forward in R&D on this genus and understand the following (i) What is the extent of research and scientific publication on different species of the genus *Melia*? And (ii) What are the main research topics for the genus *Melia* and how have these changed over time?

## Materials and methods

For this investigation, the Web of Science (WoS) database was selected due to its comprehensive coverage. The literature corpus was collected using the single keyword TC = “Melia” for the period between 1946 and 2022 [accessed on 01.12.2022]. Considering the broader nature of research publications from different disciplines, it was decided to use a single-word search rather than a string comprising all the species of the genus *Melia*. This was carried out to eliminate the variations of spelling in the specific epithet predominant in the literature^16^. Other studies of topic-specific searches have recounted the increased specificity and recovery of information^17,18^. The JabRef open-source software version 5.0 was used for duplicate searching and rectification. The search in WoS yielded 1523 scientific articles. After scrutiny for other language articles as well as book chapters, books, notes, meeting abstracts, correction, book reviews and editorial material—about 1263 literature was used in the analysis^14^ ( Figure S1). The metadata was downloaded in the BibTeX format and analyzed in R (version 4.0.1) and RStudio (Version 1.3.959) using the bibliometrix R-package (http://www.bibliometrix.org)^19^. This package has a biblioshiny tool through which importing, cleaning, and organizing bibliographic data, and for conducting various types of bibliometric analysis. This includes analysis of the citation patterns of individual papers or authors, analysis of the co-citation patterns of papers and authors, and analysis of the evolution of scientific fields over time. Further, SciMAT (version 1.1.04) was used to carry out for overlapping of keywords^20^. This open-source software has some advantages compared to other science mapping tools^21–23^. The analysis was designed with the following parameters: Unit of analysis, Words (authorRole = true, sourceRole = true, addedRole = true); Kind of network: Co-occurrence; Normalization measure: Equivalence Index; Cluster algorithm: Simple Centres (Max cluster size: 4, Min cluster size: 3); Evolution measure: Jaccard Index; Overlapping measure: Inclusion Strength.

## Results

**Figure 1:**
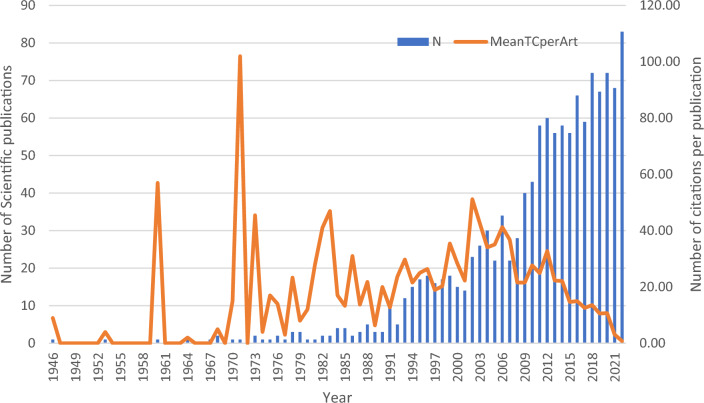
Scientific literature on genus *Melia* from 1946 to 2022.

Over 75 years from 1946 to 2022, there were 1263 scientific publications published in 561 journals from 88 different countries (Figure S2). There was an annual growth rate in publications of 5.99% which peaked after 2010. *Melia azedarach* was the most researched species (n = 1042), followed by *M. toosendan* (n = 136)*, M. dubia* (n = 68), *M. volkensii* (n = 50)*, M. azadirachta* (n = 42), *M. composita* (n = 11), *M. indica* (n = 3), *M*. *birmanica* (n = 1)*, and M. excelsa* (n = 1) (Fig. 1).

**Figure 2:**
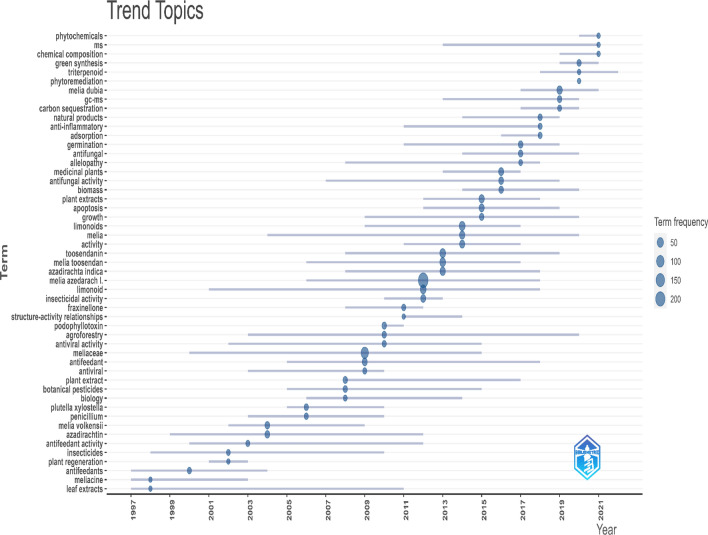
Dynamics of research topics on the basis of Authors Keywords.

The change in topics over time related to the genus *Melia* is presented in Fig. 2 as a trend topic graph—a form of scatter diagram. This longitudinal analysis points out the evolution of topics over time. This figure is the output from the consolidation of the metadata for documents retrieved from the WoS database after processing and elimination of duplicates. The y-axis lists the trending topics (based on the frequency of occurrence) with reference to the timeline (years) in the x-axis. The bubble and its size indicate the proportional occurrence of the topic; and the bar along the x-axis indicates the first and third quartiles of topic occurrences.

The reference year for a particular topic was identified using the median of the distribution for the topic over the time period considered. From the analysis, it was evident that *Melia dubia* has been on the research front in recent years. This is followed by *M. toosendan* and *M. volkensii.* It is evident that the *Melia* species were studied for their secondary metabolite constituents as well as their insecticidal and antimicrobial activity until 2010. Subsequently, the research interest shifted to growth, biomass, and germination. From 2019 onwards, new themes such as green synthesis and chemical composition emerged with the advent of mass spectroscopy (ms) and gas chromatography-mass spectrometry (gc–ms) techniques.

The thematic evolution of genus *Melia* and scientific mapping are depicted as strategic diagrams (Fig. 4). From 1946 to 2006, themes such as *Melia azedarach, agroforestry, Azadirachta indica, Spodoptera, Melia toosendan,* etc. are dominant. *Melia azedarach* is one of the key species of genus *Melia* present in every sub-period: (a) 1946–2006; (b) 2007–2014; (c) 2015–2018 and (d) 2019–2022. In a typical strategic diagram, the research theme is obtained after co-word analysis and each theme are characterized by the density and centrality values^24^. Based on the values, the research themes were categorized into four major groups (a) upper right quadrant are the motor themes (b) upper left theme are highly developed and isolated themes (c) lower left quadrant are emerging or declining themes and (d) lower left is the basic and transversal themes.

**Figure 3:**
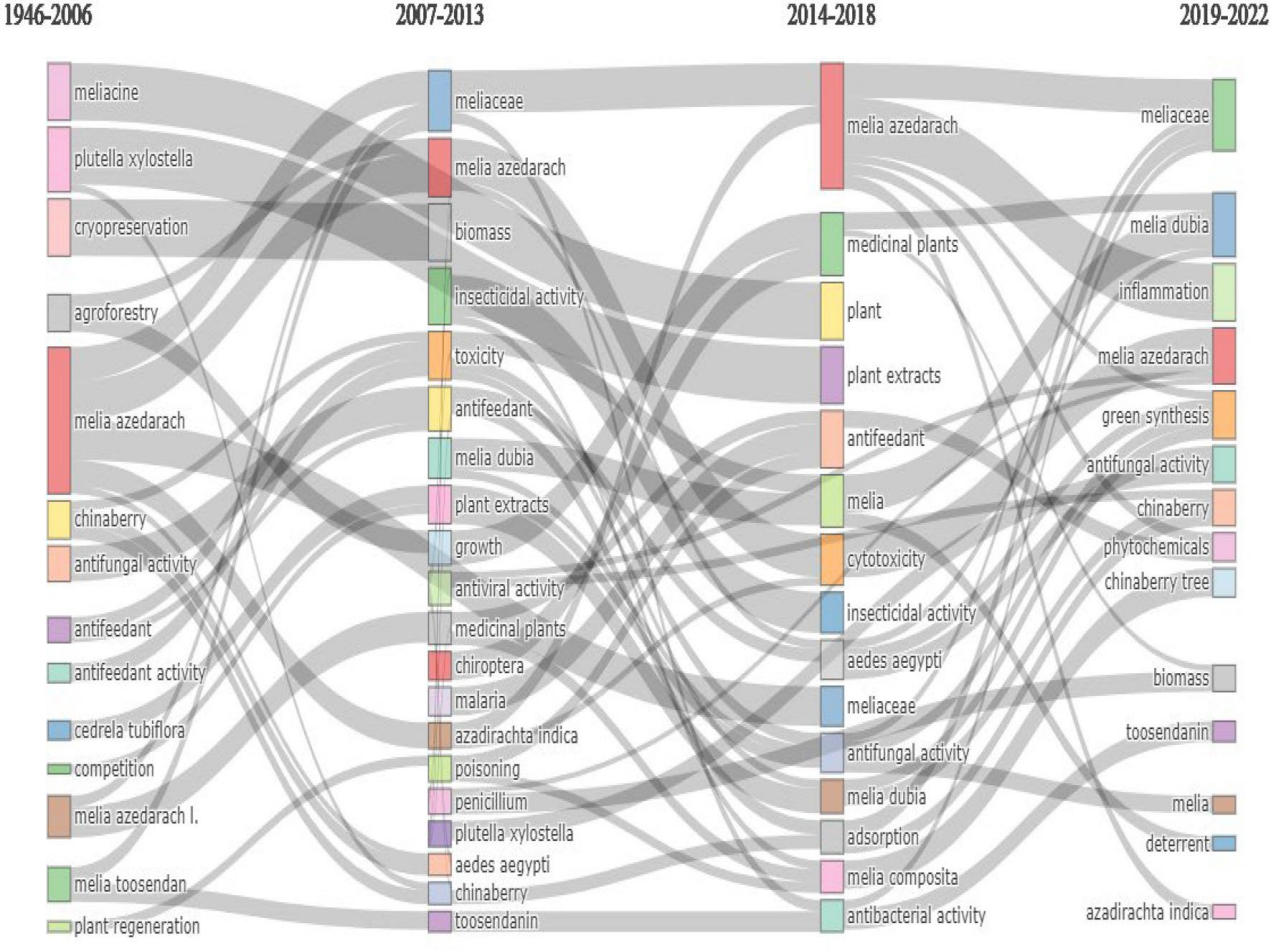
Thematic evolution of the keywords.

The thematic evolution map in Fig. 3 depicts the connection between the leading research theme in each time period (with inclusion index equal to or greater than 0.5). The most solid theme was led by the topic *Melia azedarach* in all most all subperiods having connections with almost all other research theme. Some research themes such as *Chinaberry, Insecticidal activity, Malaria, Melia dubia, Penicillium aedes egypti, Biomass, Melia composita* and *antifungal activity* had negligible connections.

**Figure 4:**
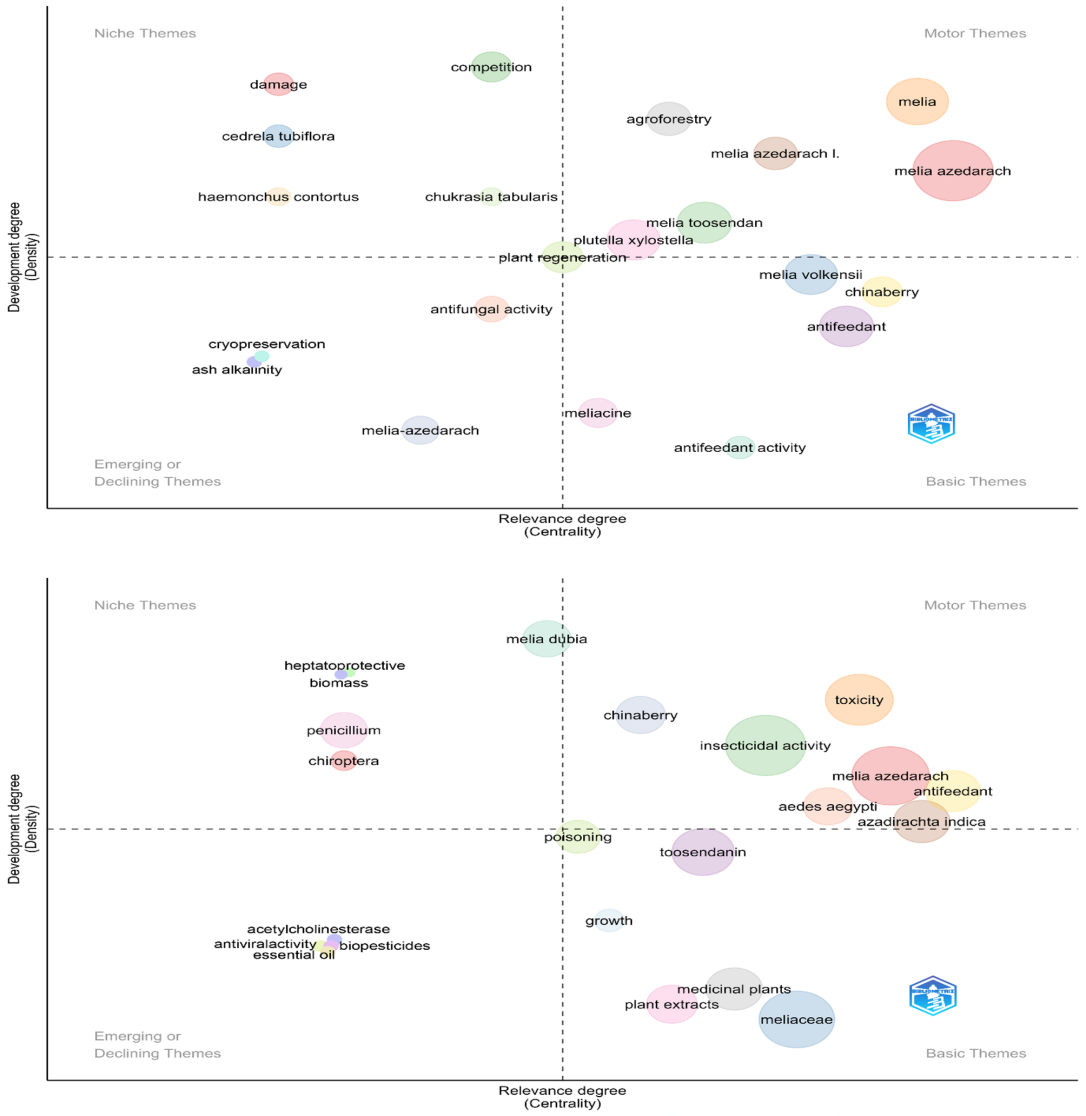

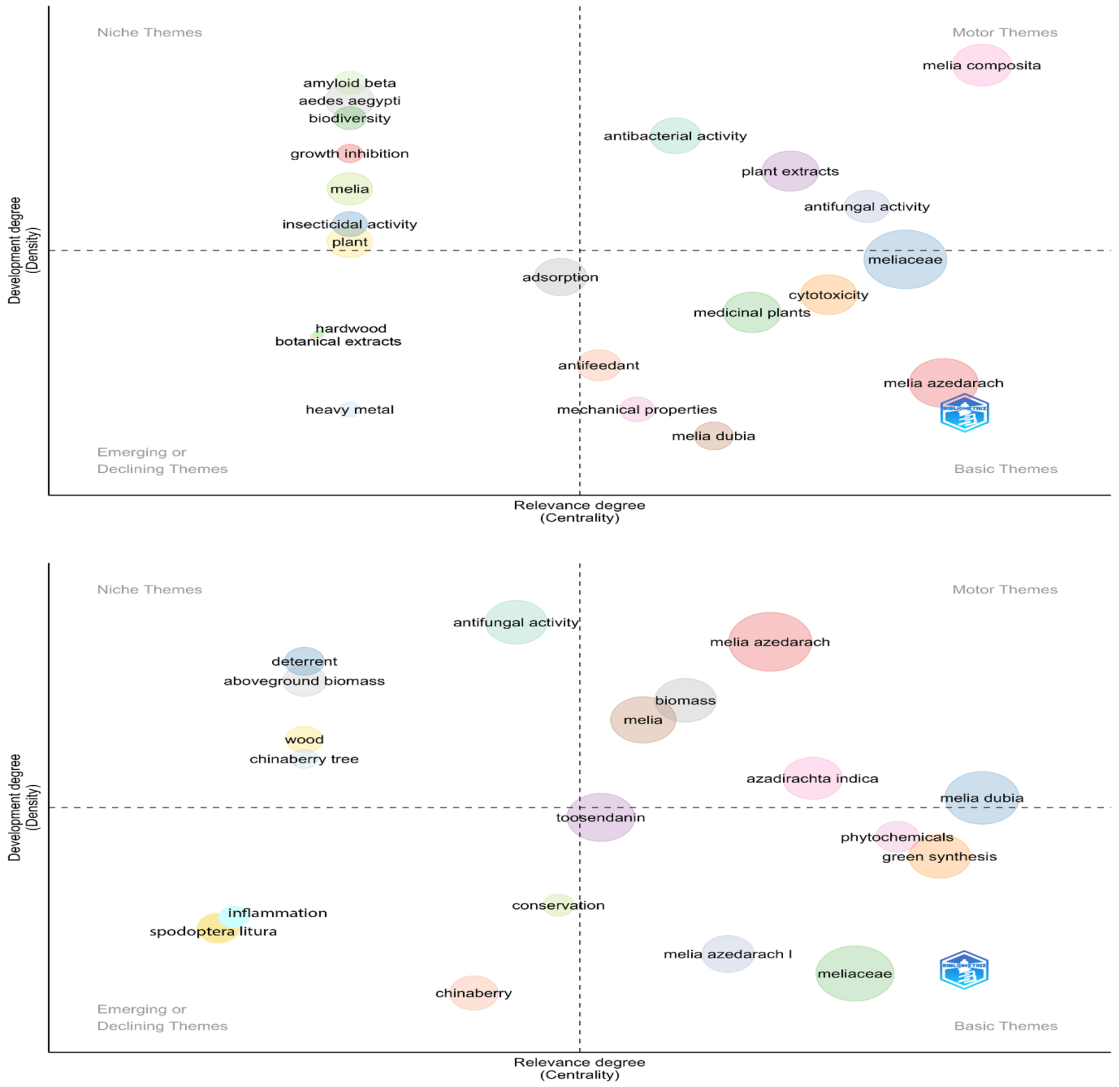
Strategic diagram for sub-period (**a**)1946–2006; (**b**) 2007–2014; (**c**)2015–2018 and (**d**) 2019–2022.

In all time periods, the theme *Melia azedarach* was persistent whereas other research themes which area botanical names includes *Melia toosendan, M. volkensii, Chukrasia tabularis* and *Cederela tubiflora* in the time period 1946–2006. It also evident that all other research theme clusters were always related to secondary metabolite components and their utility. The strategic diagrams also reveal the transition of a research theme—*melia dubia.* The theme appeared as basic theme between 2007 and 2013 and slowly transformed into motor theme by 2019–2022, with slight fluctuations. There is also evident that early research (1946–2006) on this genus was focused on utility, including *antifeedant activity* and *antifungal activity* along with possibility of growing this species under *agroforestry* and *plant regeneration* (Fig. 4)*.* In the subsequent time periods (2007–2013 & 2014–2018), the research focused more on secondary metabolites, with a slight inclination to growth and wood properties. By the end of last time period (2019–2022), growth and wood and biomass production got more attention. Overall, it is clear some species like *M. azedarach, M. volkensii, M. composita, Azadirachta indica* and *M. toosendan* were focused by the research fraternity across the world. It seems that China with about 849 publications topped the most contributing nation followed by India (n = 638).

**Figure 5:**
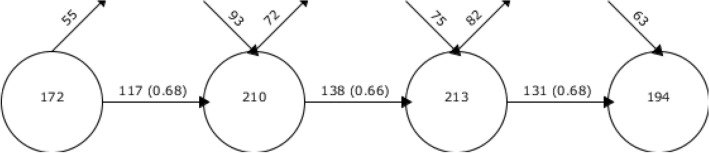
Keywords overlapping in the map of publications on Genus *Melia* (Research domain): (**a**) 1946–2006, (**b**) 2007–2014, (**c**) 2015–2018, and (**d**) 2019–2022.

The science mapping was done in SciMAT tool by grouping the keywords and is depicted in Fig. 3. During the initial period of 1946–2006, 172 keywords were found. In 2007–2014, 55 keywords did not reappear, 117 keywords were migrated and 93 new keywords were added by researchers. In 2015–2018, 72 keywords were not used and 75 new keywords were added and along with 213 keywords were used by researchers. In 2019–2022, there were 194 keywords used in publications, with about 131 keywords continued from 2014 to 2017 (Fig. 5). The steady usage of keywords over the years indicates the stagnation in the research base on Melia. Meanwhile, about ~ 60% of keywords remain unchanged over the years.

## Discussion

This study aimed to understand the research facets of the genus *Melia* due to its increasing economic importance. Scientific publications are regarded as the most reliable source of information on research topics, and these are typically accessed using keyword searches in databases, including Google Scholar. Scientific production of the genus has increased linearly over the years, with some fluctuations (Fig. 1). This genus has been studied in more than 80 countries, indicating its widespread geographical distribution and interest.

Typically, scientific names are the vital links connecting the findings of researchers in different disciplines. Unfortunately, variations in the spelling of botanical names can result in an article not being found. In our study, we came across different specific epithet spellings for the genus *Melia.* For instance, the specific epithet of *Melia azedarach* was misspelled as *azedericta, azederach, azedarch, azediarach, azedarrah, azedarack, *etc*.* Moreover, there were more than 55 species listed under the genus *Melia* in the International Plant Names Index (https://www.ipni.org/), as well as approximately 57 species listed in the WFO Plant List (https://wfoplantlist.org/plant-list). However, only three species are listed as accepted: *M. dubia, M. azedarach* and *M. volkensii.* However, in our research, we could see the predominant usage of the synonyms of *M. azedarach* with different other species. For instance, *M. toosendan,* a synonym of *M. azedarach*, is regarded as a separate individual species in China^25,26^. Similarly, *M. composita* is a synonym for *M. dubia*^27^*,* but it is sometimes treated as an independent species in India^28^. Even *M. dubia* was regarded as a synonym for *M. azedarach*^29^ until Sivaraj et al. (2018) proved them to be two different independent species. These misconceptions and renamings may not be unique to the genus *Melia*. The issue raises serious concern because some *Melia* species are important in ethnobotanical and ethnoveterinary practice, while others are also reported to be toxic^12,31^. This calls for work to clarify the taxonomy of the genus.

Scientific names allow us to cross-reference information about organisms globally. However, variations in the spelling of scientific names greatly diminish the ability to interconnect data. Such variations may include abbreviations, annotations, misspellings, etc^12^. Our concerns grew are more related to variations in scientific names that can hamper the use of approaches such as text mining, systematic review, metanalysis, and research weaving^14,32^.

It is concluded that the following species are the most researched species in the genus *Melia* based on the number of citations: *M. azedarach, M. toosendan, Melia dubia,* and *M. composita.* The trend topics also pointed out a similar list of species (Fig. 2). We also found papers on *M. indica*, *M. birmanica*, and *M. excelsa* on topics such as metabolite constituents and wood properties. However, these publications were not cited. Most of them were published before 2000, except for Iswanto et al. (2010), which dealt with wood properties of *Melia excelsa.* It seems that *Melia excelsa* is a synonym for *Azadirachta excelsa,* but this synonym is rarely used in scientific literature.

The relevant main topics and their changes over time with respect to the genus *Melia* are assessed based on trend topics (Fig. 2), a thematic evolution map (Fig. 3), and a strategic diagram (Fig. 4). These were based on coword analysis performed with the Louvain algorithm due to its good performance in terms of modularity and processing time^34^. For a typical thematic evolution map, the total publications will be divided into subperiods. In our analysis, given the vast differences in the levels of production over time, the overall publications were grouped into four major timelines such that the average number of publications in a single time period was ~ 300 scientific papers. Combined insights from science mapping were used to measure the development of the specific research theme in a conceptual way for a specific time period.

Earlier research on the genus was focused on its medicinal and repellent properties. For instance, one of the oldest publications on the genus *Melia* is on the exploration of the repellent properties of its two species^35^. *Melia azedarach *leaf extract might have antiviral properties, which was studied in the late 1990s^36^. The possibility of using it for controlling specific insects, such as the diamondback moth (Plutella xylostella), triatomine bugs, the variegated cutworm (*Peridroma saucia*), the maize weevil *(Sitophilus zeamais)*, many, other insects, and some fungal diseases, was also explored. These initial investigations on insecticidal and antifeedant activity encouraged researchers to explore more at the biochemical and genome levels in more recent years^37^ (Fig. 2). *Melia azedarach* and *Azadirachta indica* have both been well-studied secondary metabolites, and both are in the Meliaceae family. *Melia azedarach* being the most detailed studied species of the genus *Melia*. A separated analysis of metadata of 1042 publications revealed that similar trend in the change in topics (Figure S3). Wood usage was not a focus of research for the genus *Melia* until the early 2000s^38^ (Fig. 4d). In the last few years, the entire focus of *Melia* research has been on its usage as a potential wood-yielding species. This has led to research on its germination, reproduction, growth and biomass production. We hypothesize that the increased wood demand and trade has urged stakeholders to explore suitable alternative species for wood production^39^. In addition, researchers have worked on some fast-growing trees, such as Eucalyptus, Popular, and Casuarina, and have stumbled upon the fast-growing *Melia* and ventured into this genus. *Melia dubia* and *M. composita* have proven wood qualities, unlike *M. azedarach,* which is regarded as an invasive plant in the USA and some parts of Africa^40,41^. Nevertheless, *M. azedarach* is noted for paper and pulp^42^. Recently, there has been increased research on the green synthesis of nanoparticles related to *Melia,* which hints at new potential for this genus.

The potential limitation of this study will the exclusive usage of WoS database alone. Also, the non-inclusion of articles in other languages is a limitation to this study. However, we made a narrative review of most publication to avoid bias in judgement which is quoted as a limitation for research weaving based studies (Ekundayo and Okoh, 2018). The wider coverage in WoS reduces the “indexer effect”, thus making the significance of the findings. It is also pertinent to point out that the findings on shift in research trends on genus *Melia* will be revealed even after inclusion of publications from other database like Scopus.

Overall, the genus *Melia* is naturally distributed across many countries and has been studied in different parts of the world for various uses. *Melia* has been studied by a broad group of researchers, but the identification, taxonomy and nomenclature of some species of Melia remain unknown. The taxonomic uncertainty of several species continues in this genus, which could have a long-term effect on utility research and development with this genus. This in turn can have a cascadal effect on the comprehension and field utilization, and genetic resource maintenance of the species. Although the genus was used for its secondary metabolites in the early days, in recent years, research has focused more on wood and timber values. This shift in the research focus is expected to impact the wood supply at the global scale owing to its fast growth and the possibility of cultivation over a wide geographical range.

Our analysis indicate that members of the genus *Melia* have multipurpose utility apart from the ethnobotanical uses and current research on certain aspect like plantation forestry as well as agroforestry on this genus will have an impact on the wood demand–supply. Specially in India, the India State Forest Report (2021) mentions the role of Trees Outside Forests (TOF) in meeting the wood/timber demand of the country. Predominately these TOF are in farm lands and it is dominated with short rotation trees. In this juncture, this study points out the species-specific zonation for their progressive adaptation in different bio-geographical region. For instance, the *M. volkensii* is suited for short rotation plantation forestry in Africa and *M.dubia* is well suited for agroforestry in Indian sub-continent. There are potential chances for the inter-continental shift of this genus (genetic resources) for wider utility following the protocols and institutional arrangements. Three decades earlier, *Tectona grandis* was introduced in African and South American countries which have performed well and augmented the farm income. Similarly, the *Santalum album* in Australia, likewise, the Melia sp. has such potential. Also countries across the globe want to augment the area under forest and tree cover. Apart from tree plantation drive, the adoption and promotion of trees in farm lands can be more enhancive as it has happened in the Asia countries. In Southeast Asia, 77.80% of all agricultural land is under agroforestry practice, while in East Asia, it is 50.50%; in South Asia 27%, and in Northern and Central Asia, 23.60%. Apart from the other reasons, the exploration of different tree species and its cultivation has enabled it. Atleast, *Melia dubia* having the fullest potential can be deployed for diversified agroforestry to provide food, fodder, fuel and income to the tropical farming communities.

### Supplementary Information

Supplementary Figures.

## Data Availability

The datasets used and/or analysed during the current study available from the corresponding author on reasonable request.
