# Supplementary material for: Exploring research trends and priorities of genus Melia
Source: Sci Rep. 2024 Mar 15;14:6265. doi: 10.1038/s41598-024-53736-3 (PMC10943012; doi:10.1038/s41598-024-53736-3)
Supplement: Supplementary file 1 — Supplementary Figures. [file 41598_2024_53736_MOESM1_ESM.docx]

**Supplementary Figure S1: The workflow diagram based on PRISMA guidelines adopted for this study**


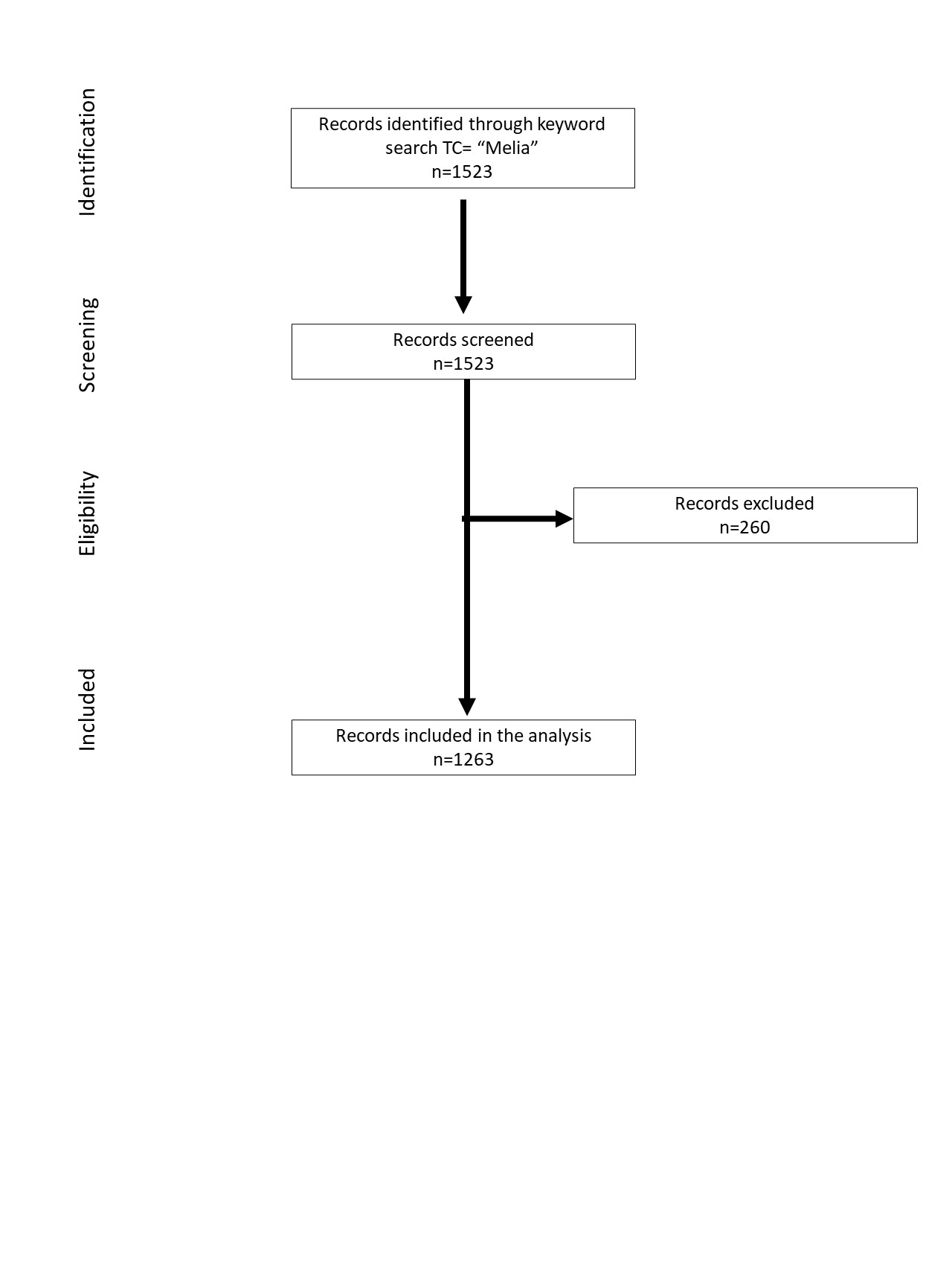


Metadata of publications excluded in the analysis include publications from other languages [Portuguese (31), Spanish (14), German (11), Chinese (4), Japanese (4), French (2), Turkish (2), Russian (1)]

**Supplementary Figure S2: The geographical distribution of publications based on the corresponding author**


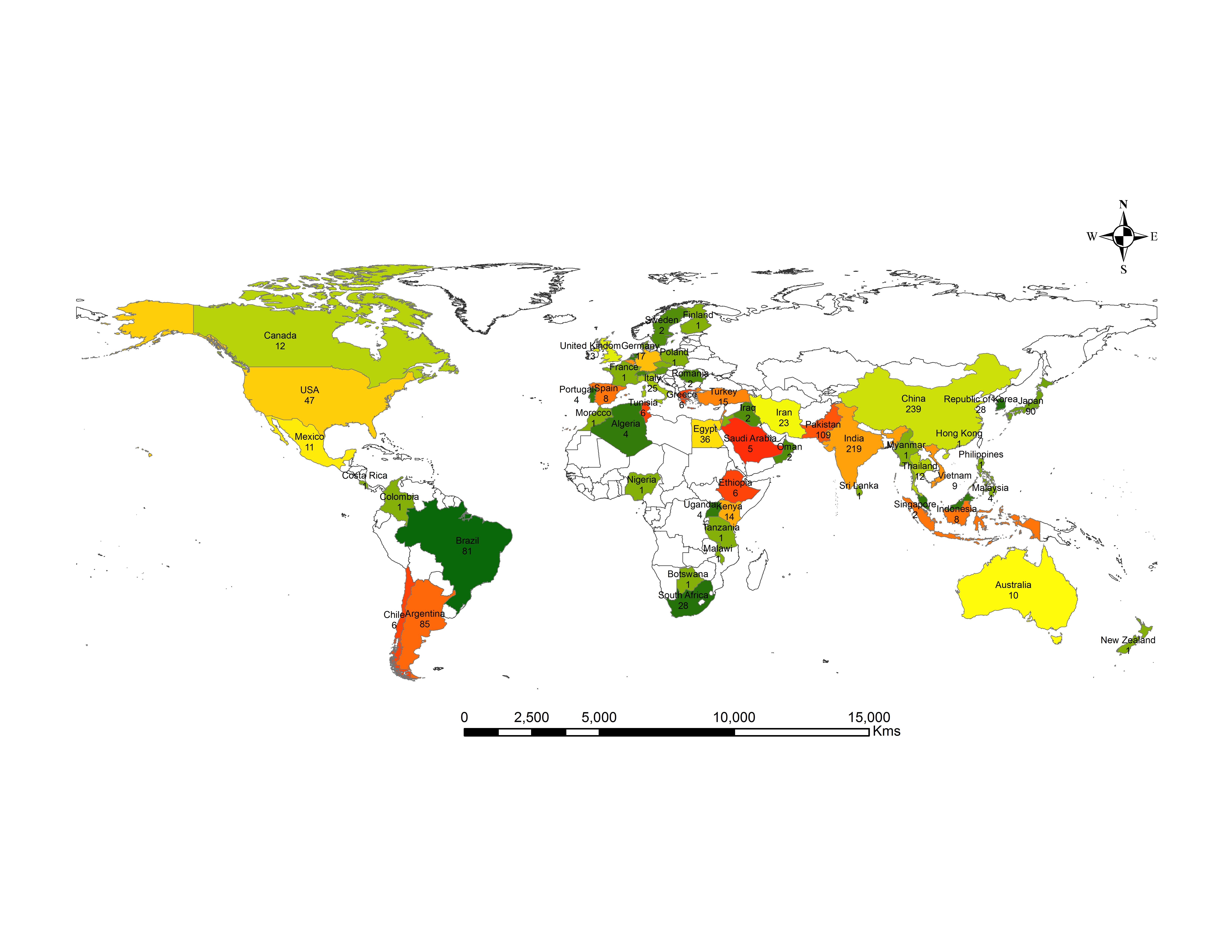


**
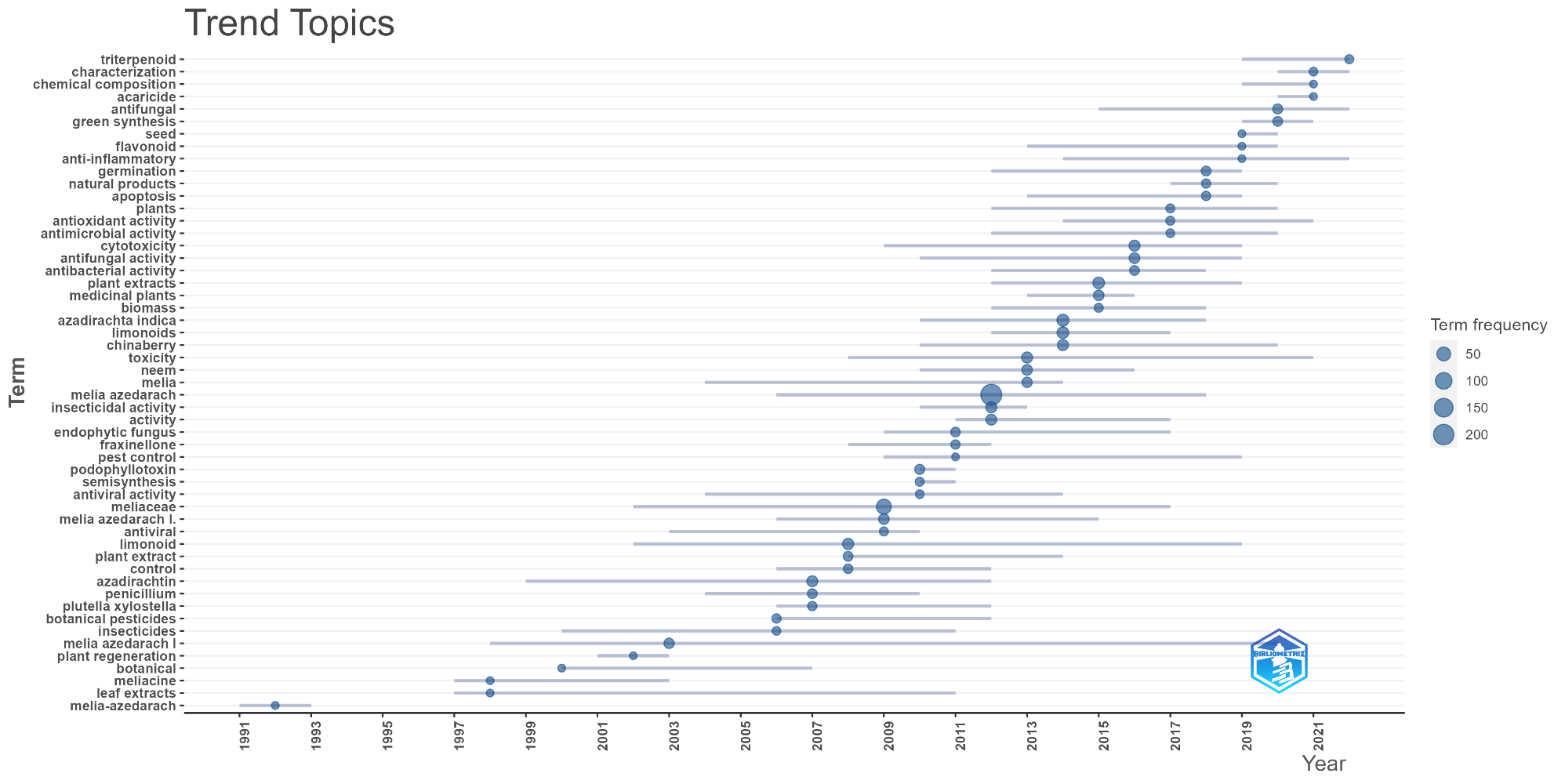
**

**Figure S3. Dynamics of research topics on the basis of Authors Keywords for the literature of *Melia* *azedarach***
